# Predictors of Patient Satisfaction with Tertiary Hospitals in Korea

**DOI:** 10.1155/2015/749754

**Published:** 2015-02-05

**Authors:** Hye-Sook Ham, Eun Hee Peck, Hee Soo Moon, Hye-A Yeom

**Affiliations:** ^1^Nursing Department, Seoul St. Mary's Hospital, Seoul 137-701, Republic of Korea; ^2^College of Nursing, The Catholic University of Korea, Seoul 137-701, Republic of Korea

## Abstract

This study examined the general and system-related predictors of outpatient satisfaction with tertiary health care institutions in Korea. A cross-sectional descriptive study design was employed. The subjects were 1,194 outpatients recruited from 29 outpatient clinics of a university medical center in Korea. Measurements included 5 outpatient service domains (i.e., doctor service, nurse service, technician service, convenience, and physical environment of facility) and patient satisfaction. Of the five domains, nurse service was the domain with the highest mean score (*M* = 4.21) and convenience was the domain with the lowest mean score (*M* = 3.77). The most significant predictor of patient satisfaction was the constructs of convenience (*β* = 0.21). The results of this study suggest that the concept of patient satisfaction with health care institutions in modern hospitals reflects an integrative process that includes not only the concerned health care personnel but also improved convenience such as user-friendly reservation system and comfortable waiting areas.

## 1. Introduction

Improving patient satisfaction is a key element in strategies for improving the long-term economic viability of health care institutions. Patient satisfaction, defined as the client's overall positive evaluation of the health care services [1], largely influences health care provider choice in health care sector [2–4]. Patients satisfied with the health care services of a particular hospital may tend to visit the institution consistently and maintain beneficial relationships with health care providers. Such patients are also likely to show high compliance with their prescribed treatment plans and maintain positive relationships with medical staff with less chance of legal sue for practice, which may ultimately facilitate their collaboration with health care providers [5–7]. For these reasons, hospitals providing tertiary care services regard customer satisfaction as a crucial determinant of institutional viability and make efforts to respond flexibly to changing health care environments and the public's health care needs.

Patient satisfaction is evaluated on the basis of both provider- and client-focused aspects. While provider-focused aspects refer to the provision of sound medical skills, client-focused aspects are centered on the extent to which the patients feel their needs and expectations are being met during the provision of health care services [8–10]. Previous researches have reported that gaps between these aspects exist [11, 12], suggesting that identifying factors affecting perceived customer satisfaction is critical for providing quality care.

Knowing the predictors of patient satisfaction and loyalty is crucial in the present health care market in which meeting patients' needs is one of the most important marketing strategies and indicators of hospital competency. In outpatient clinics, the health costs spent by outpatients have been increasing gradually, mainly due to prevalent chronic conditions such as hypertension, diabetes, arthritis, and renal diseases [13]. Specifically, in Korea, whereas the economic role of outpatient clinics has been gaining importance with an increasing volume of visitors in tertiary care hospitals (1,056 outpatients per day) [14], research on patient satisfaction has focused mainly on hospitalized or primary care patients while making limited focus on outpatient settings. Therefore, this study aimed to examine the general and system-related predictors of outpatient satisfaction with health care institutions in order to provide information for effective outpatient health care services in tertiary hospitals in Korea.

## 2. Method

### 2.1. Design and Sample

A cross-sectional descriptive study design was employed. The subjects were a total of 1,194 outpatients recruited from 29 outpatient clinics of a university medical center with over 1,000 beds, located in Seoul, Republic of Korea. This sample size was approximately 20% of the total number of outpatients of the hospital per day and was estimated based on a significance level of 0.01, a power of 0.80, and an effect size of 0.10 [15]. A proportional random sampling technique was used to increase the representative nature of the sample for the target population. With a target number of subjects of 1,000, the survey volume allocated to each outpatient unit was determined on the basis of its relative proportion to the entire sample in terms of the average monthly number of outpatients. To increase external validity, only patients with a registration number ending in an odd digit were recruited for participation. All participants were Koreans who met the following inclusion criteria: (1) age above 18 years, (2) ability to communicate verbally in Korean, and (3) ability to understand the aim of the study and to provide written consent for study participation.

### 2.2. Measurement

#### 2.2.1. General Characteristics

General characteristics included sex, age, residential region, religion, employment status, revisit of outpatient clinic, type of doctor, total length of being an outpatient of the institution, and experience of use of other tertiary hospitals (Table 1).

#### 2.2.2. Scale Development Process

Due to the scarcity of outpatient-specific health care evaluation tools for Koreans in a tertiary care setting, the instrument for evaluating the quality of health care services in this study was developed by the investigators in 3 steps. In the first step, outpatient-specific health care service questions were designed based on the theoretical framework proposed by Donabedian [16] in regard to essential components quality health care, a customer satisfaction survey of a domestic client service department of the hospital, and the National Customer Satisfaction Index [17]. A panel of expert clinicians consisting of 1 nursing department team manager, 2 unit managers, and 23 duty-in-charge nurses in outpatient clinics participated in the development of the initial version of the questionnaire. A total of 88 questions in 11 constructs were developed as the primary version of the outpatient health care service evaluation scale for this study.

Second, a pilot test of the questionnaire was conducted to examine its feasibility and applicability for Korean outpatients in a tertiary care setting. A convenient sample of 200 outpatients selected from 4 outpatient departments (i.e., Endocrinology, Orthopedics Surgery, Obstetrics and Gynecology, and Pediatrics) completed the questionnaire. No single item of the primary version of the questionnaire was omitted or questioned by the subjects, supporting the feasibility of the scale to Korean outpatients in a tertiary hospital.

The third and last step was the evaluation of content validity. The content validity was verified by a panel of experts consisting of 1 team manager, 2 nurse managers, 1 nursing professor, 1 research assistant, and 1 outpatient staff nurse. The content validity of each item was evaluated using a visual analog scale of scores 0-1, where 0 and 1 indicated no and full content validity, respectively. Items with a score exceeding 0.80 of 1.0 were regarded as having content validity [18]. Constructs regarded as overly institution-specific or conceptually irrelevant to outpatient services, such as pastoral and admission services, were also excluded from the primary version of the instrument. This scale refinement process resulted in the exclusion of 46 items and 6 service domains. As a result, the final version scale used for this study contained 5 service domains with 42 items that include 41 items representing the quality of outpatient services and a single item indicating patients' service satisfaction.

#### 2.2.3. Service Factors

The service factors consisted of 5 constructs including doctor service (8 items), nurse service (10 items), technician service (11 items), convenience (6 items), and facility/physical environment (6 items). Since each of these domains represents unique role or function requiring domain-specific interventions, the mean scores for each domain were estimated separately. The higher the mean score, the higher quality health care the outpatient experienced in each domain.

The domain of doctor service consisted of 8 items including friendly manner, explanation of diagnostic results and treatment process, careful listening, respect and courtesy, attentive responses to the patient's needs, sufficient time spent on the patient, thorough history taking, and professional appearance. The Cronbach alpha reliability of the construct of doctor service was 0.93.

The domain of nurse service contained 10 items including friendly manner, explanation of the outpatient care process at check-in, careful listening, respect and courtesy, attentive responses to the patient's needs, explanation of the anticipated waiting time, explanation of the reason for delayed care, provision of postcare instructions at check-out, protection of privacy, and professional appearance. The Cronbach alpha reliability of the domain of nurse service was 0.80.

The domain of technician service contained a total of 11 items including the technician's friendly manner, explanation of the process of treatment or diagnostic procedure, careful listening, respect and courtesy, attentive responses to the patient's needs, provision of waiting list on board, explanation of the anticipated waiting time, explanation for delays, provision of postprocedure instructions, protection of privacy, and professional appearance. The Cronbach alpha reliability of the construct was 0.90.

The domain of convenience consisted of 6 items including timely check-in and check-out processes, convenient reservation system for clinic appointments, convenient reservation system for treatments/procedures, provision of outpatient care on time, acceptable waiting time, and time-efficient on-site payment system. The Cronbach alpha reliability of the construct was 0.76.

The domain of facility/physical environment contained 6 items including overall cleanliness of the facility, comfortable waiting area, clean restroom, access to amenities (e.g., cafeteria and bank), parking/access to public transportation, and easily recognizable signals for directions. The Cronbach alpha reliability of this construct was 0.91.

#### 2.2.4. Patient Satisfaction

Patient satisfaction was measured by a single question: “Are you satisfied with overall outpatient health care services of this hospital?” Participants responded on a 5-point, Likert scale, with “very satisfied” scored as 5, “satisfied” as 4, “moderate” as 3, “dissatisfied” as 2, and “very dissatisfied” as 1. Higher scores indicated higher levels of patient satisfaction. The reliability of the questionnaire was not examined since the scale included only a single item, making measuring internal consistency inappropriate.

### 2.3. Data Collection Procedure

Upon approval from the institutional ethics review board of the study site (IRB number KC11QISI0347), proportional volumes of survey questionnaires based on the average monthly numbers of outpatients were distributed to the 29 outpatient units of the hospital. Trained outpatient nurses then screened the eligibility of outpatients on the basis of the inclusion criteria and presented the research aim and overall survey procedure with the assurance of confidentiality and protection of privacy to eligible subjects. Self-administered questionnaires were distributed to those who agreed to participate. Partial assistance for completing the survey questionnaire by a family caregiver was allowed for those physically challenged and/or aged over 65 years. After the anonymous questionnaire was completed, each questionnaire was placed into a sealed box located at the marginal side of the nursing station of each outpatient unit. The data were collected in June 2011 for the pilot survey and from July to August 2011 for the original survey of this study.

### 2.4. Data Analysis

Data were analyzed using SPSS version 12.0 for Windows. Descriptive statistics were used to describe the general characteristics and levels of patient satisfaction of the sample. *t*-tests were used to examine the associations of patient satisfaction with general characteristics including sex, residential region, religion, employment status, revisit of outpatient clinic, type of doctor, and use of other tertiary hospitals. ANOVA was used to examine the associations of patient satisfaction with age, religion, and total length of being an outpatient of the institution. Scheffé's test was used as a post hoc test to determine differences among subgroups based on demographic characteristics with respect to patient satisfaction. Simultaneous multiple regression analyses were performed to examine service factors and individual variables predicting patient satisfaction.

## 3. Results

### 3.1. General Characteristics

Of the study participants, 56% were women and 37% were men. Participants were most likely to be older than 60 years of age (22.2%), residing in Seoul (53.9%), and Roman Catholic (27.7%). The majority of the subjects (66.8%) were employed. Most subjects were revisiting patients (65.8%) and sought care from physician specialist (59.0%). Regarding the total length of the use of hospital services, a period of 2–5 years was the most common (20.6%) followed by a period of less than 1 year (15.2%). The majority of the subjects (71.9%) reported having experiences of visiting other hospitals, whereas 21.0% had no previous experience of using health services of other tertiary care institutions (21.0%) (Table 1).

### 3.2. Patient Satisfaction and Service Factors

The mean overall score of outpatient satisfaction was 3.98 of 5 (SD = 0.75). There were no associations between the general characteristics of the subjects and their levels of satisfaction with the hospital (Table 1). Mean scores for five domains were 4.21 for nurse service, 4.20 for doctor service, 4.08 for facility/physical environment, 3.94 for technician service, and 3.77 for convenience. Of individual items of the domain of nurse service, “explanation of outpatient care process” was the one with the highest mean score (4.35), and “explanation for delays” was the one with the lowest score (3.94).

### 3.3. Predictors of Patient Satisfaction

Analysis of the standardized beta, which indicates the magnitude of correlations of the 5 service domains with patient satisfaction, showed that convenience was the most significant predictor of patient satisfaction (Table 2). An examination of the standardized beta by the individual items of the domains convenience showed that convenient check-in and check-out processes had the greatest impacts on patient satisfaction in the domain of convenience (*β* = 0.21), followed by convenient reservation system for treatment/procedure (*β* = 0.21) and acceptable waiting time (*β* = 0.15).

The most significant predictor of patient satisfaction in the domain of nurse service was professional appearance (*β* = 0.14), followed by careful listening (*β* = 0.13) and explanations for delays (*β* = 0.12). In the domain of doctor service, the most significant predictor of patient satisfaction was respect and courtesy for the patient (*β* = 0.21), followed by thorough history taking (*β* = 0.19) and sufficient time spent with the patient (*β* = 0.12). Of the individual items of the technician service domain, the most significant predictor of patient satisfaction was the technician's friendly manner (*β* = 0.16), followed by respect and courtesy for the patient (*β* = 0.16) and explanations for delays (*β* = 0.15). The most significant predictor of patient satisfaction in the domain of the facility/physical environment was the presence of a comfortable waiting area (*β* = 0.27) followed by maintenance of clean restrooms (*β* = 0.24) and parking and access to public transportation (*β* = 0.19).

## 4. Discussion

This study examined the levels and predictors of outpatients' satisfaction with health care services in an outpatient setting of a tertiary care institution in Korea. The domain of outpatient services consisted of 5 constructs in this study, including doctor service, nurse service, technician service, convenience, and facility/physical environment. The mean score of overall level of outpatient satisfaction was 3.98 of 5. This level of satisfaction is lower than 4.10 of 5, reported by Hwang and Son [19], but comparable to 5.83 of 7, reported by Rho and Oh [20]. However, direct comparison among these findings was difficult due to the use of different scales, suggesting the need for future replication studies using a consistent measurement.

Interestingly, the service domain that explained outpatient satisfaction most significantly was the construct of convenience. This result is not consistent with the findings of a previous study reporting that doctor service accounts for patient satisfaction most significantly [21] but is consistent with the view that patients are significantly affected by facilities and convenience such as short waiting time and satisfaction with the waiting environment [22, 23]. Among the individual items of the convenience domain, convenient check-in and check-out processes were the most significant predictor of patient satisfaction, supporting the importance of system management characteristics such as reduced waiting times and efficient billing systems for understanding the needs of outpatients and increasing their satisfaction for health care services in tertiary care hospitals.

The most significant predictor of patient satisfaction in nurse service domain was professional appearance. The importance of a professional appearance has been specifically reported to be correlated with patient satisfaction in previous research [24], indicating that nurses' professional image in outpatient settings conveys a positive impression to patients. Friendly manner of the staff affected patient satisfaction most significantly in the technician service domain, which is consistent with the finding of a previous study that showed that a good attitude of the staff is an essential component of the successful administration of health care [25, 26]. It should be strongly emphasized that friendly communication of the hospital staff may enhance customer satisfaction in the health care industry.

In the present study, there were no associations between general characteristics and patient satisfaction, which is not in accord with the findings of a previous study that female gender and older age are associated with increased patient satisfaction [27]. Further replication research should be conducted to examine demographic predictors of outpatient satisfaction and to explore whether marketing strategies for providing quality health services should focus more on the creation and maintenance of effective system management rather than targeting specific demographics.

Major limitations of this study are the lack of a causal relationship among variables by the use of a cross-sectional design and limited generalizability of the findings due to the collection of the data from one tertiary hospital in Korea. Longitudinal follow-up or use of advanced statistics techniques such as casual modeling technique (i.e., structural equation modeling) should be considered in future studies to analyze the causal relationships among variables used in this study. There is also an inherent difficulty in using the investigator-developed scale to assert firm validity of the measurements, suggesting the need for further testing of psychometric properties of the scale in future studies. Nevertheless, the findings of this study may help clinicians and researchers capture the current trend of outpatient needs by providing evidence on core components of patient satisfaction in outpatient settings in tertiary hospitals in Korea.

## 5. Conclusion

The results of this study suggest that the service domain that explained outpatient satisfaction most significantly was the construct of convenience. From a managerial standpoint, it is concluded that improving convenience via user-friendly reservation systems and comfortable waiting areas, as well as professional appearance, and provision of ample information about the outpatient care process by nurses can be effective ways of increasing outpatient satisfaction with tertiary care hospitals in the future. Scholarly implications also include that further theory-based research should focus on creation of an outpatient specific quality health care model demonstrating detailed pathways to increase patient satisfaction in the outpatient settings of tertiary care hospitals.

## Figures and Tables

**Table 1:** General characteristics of study participants (*N* = 1194).

Characteristics	*n* (%)	Outpatient satisfaction			
		Mean	SD	*t* or *F*	*P*
Sex					
Male	441 (36.9)	4.04	.74	1.49	.14
Female	671 (56.2)	3.97	.72		
Age (year)					
18–20	16 (1.3)	3.99	.81	.94	.48
21–29	91 (7.6)	4.07	.79		
30–39	194 (16.2)	3.95	.67		
40–49	221 (18.5)	4.01	.71		
50–59	231 (19.3)	4.00	.73		
60–69	158 (13.2)	4.12	.69		
70–79	81 (6.8)	4.01	.81		
≥80	25 (2.1)	4.17	.64		
Residential region					
Capital city (Seoul)	643 (53.9)	3.98	.73	.20	.84
Noncapital city	393 (32.9)	3.97	.78		
Religion					
Protestant	268 (22.4)	3.97	.69	1.17	.32
Buddhist	124 (10.4)	4.05	.78		
Roman Catholic	331 (27.7)	4.06	.73		
None	290 (24.3)	3.95	.74		
Other	46 (3.9)	4.00	.74		
Employment status					
Yes	700 (58.7)	4.04	.79	.67	.80
No	397 (33.2)	3.96	.75		
Revisit of outpatient clinic					
New patient	87 (7.3)	4.07	.74	1.22	.22
Revisit patient	786 (65.8)	3.97	.73		
Type of doctor at visit					
Specialist	705 (59.0)	3.99	.73	−.65	.52
Generalist	87 (.3)	4.05	.60		
Total length of being an outpatient of the institution (year)					
≤1 year	182 (15.2)	3.98	.71	1.50	.20
2–5 years	246 (20.6)	3.93	.73		
6–10 years	143 (12.0)	4.04	.76		
10–20 years	107 (9.0)	4.11	.72		
≥21 years	61 (5.1)	3.93	.71		
Experience of use of other tertiary hospitals					
Yes	858 (71.9)	3.99	.71	−.25	.81
No	251 (21.0)	4.00	.79		

*Note*. Missing values are not included in the table.

**Table 2:** Predictors of outpatient satisfaction.

Service domain	*B*	*t*	*P*	Adjusted *R* ^2^	*F*	*P*
Convenience	.26	9.39	<.001	.58	294.21	<.001
Doctor service	.25	7.71	<.001			
Technician service	.16	4.76	<.001			
Facility/physical environment	.15	5.40	<.001			
Nurse service	.11	3.23	<.001			

*Note*. Missing values are not included in the table.
